# CA 15.3, Metastatic Burden, and Overall Survival in Hormone Receptor-Positive/HER2-Negative Advanced Breast Cancer: Real-World Evidence from Peru

**DOI:** 10.3390/biomedicines14091930

**Published:** 2026-08-28

**Authors:** Guillermo Valencia, Patricia Rioja, Jessica Meza, Alexandra Saavedra, Claudia Castillo, Armando Sánchez, Raúl Mantilla, Josué Bravo, Henry L. Gomez, Marcos Heredia, Olenka Peralta, Miguel Chirito, Connie Rabanal, Karina Aliaga, Zaida Morante, Bruno Muñante, Hugo Fuentes, Carlos Munive, Carlos Castañeda, Silvia Neciosup, Tatiana Vidaurre

**Affiliations:** 1 Department of Medical Oncology, Instituto Nacional de Enfermedades Neoplásicas (INEN), Lima 15038, Peru; patyriojavi@gmail.com (P.R.); jessica.g.11ml@gmail.com (J.M.); ale6936macarena@gmail.com (A.S.); claudiacc2697@gmail.com (C.C.); sanchez911dac@gmail.com (A.S.); marcos.heredia.100.vallejos@gmail.com (M.H.); olenka_p5@hotmail.com (O.P.); miguelchirito10@gmail.com (M.C.); connie.rabanal@hotmail.com (C.R.); karinamayraaliagallerena@gmail.com (K.A.); zmorante@gmail.com (Z.M.); dr.bruno.munante@gmail.com (B.M.); carlosmunive@hotmail.com (C.M.); carloscastanedaaltamirano@yahoo.com (C.C.); silvianeciosup@yahoo.com (S.N.); tatiana.vidaurre@gmail.com (T.V.); 2 Carrera de Medicina Humana, Facultad de Ciencias de la Salud, Universidad San Ignacio de Loyola (USIL), Lima 15024, Peru; 3 Department of Research, Instituto Nacional de Enfermedades Neoplásicas (INEN), Lima 15038, Peru; raulvicente.mantillaquispe@gmail.com; 4 Faculty of Medicine, Universidad Peruana Cayetano Heredia, Lima 15102, Peru; drbravoespinoza123@gmail.com (J.B.); hgomezmoreno@gmail.com (H.L.G.); 5 Faculty of Medicine, Universidad de Piura (UDEP), Piura 15074, Peru; fuenteshugo1@gmail.com; 6 School of Health Science, Universidad Científica del Sur (UCSUR), Lima 15067, Peru

**Keywords:** advanced breast cancer, hormone receptor-positive, HER2-negative, CA 15.3, overall survival, Peru

## Abstract

**Background/Objectives**: Serum cancer antigen 15.3 (CA 15.3) is frequently measured in advanced breast cancer, but its prognostic significance after adjusting for established clinical factors remains incompletely characterized in Latin American populations. We evaluated the association of baseline CA 15.3 with metastatic burden and overall survival (OS) in hormone receptor-positive/HER2-negative advanced breast cancer and descriptively explored baseline/follow-up patterns. **Methods**: We retrospectively evaluated 127 women treated at a Peruvian public oncology institution between 2018 and 2024, with CA 15.3 levels ≥32.4 U/mL being considered elevated. Baseline associations were assessed using multivariable logistic regression, and OS was evaluated using Kaplan–Meier estimates and Cox proportional hazards regression adjusted for age, ECOG performance status, de novo versus recurrent disease, luminal subtype, first-line treatment, number of metastatic sites, and metastatic site. **Results**: Baseline CA 15.3 was elevated in 41/127 patients (32.3%) and was independently associated with postmenopausal status (OR: 3.27; 95% CI, 1.33–9.03; *p* = 0.014) and ≥2 metastatic sites (OR: 2.72; 95% CI, 1.24–6.10; *p* = 0.013). Median OS was 55 months (95% CI, 43–78), while elevated baseline CA 15.3 was associated with shorter OS (41 vs. 65 months; log-rank *p* = 0.024) and remained independently associated with shorter OS after multivariable adjustment (adjusted HR: 3.42; 95% CI, 1.63–7.19; *p* = 0.001). **Conclusions**: Elevated baseline CA 15.3 was associated with greater metastatic burden and shorter OS, and baseline/follow-up patterns were exploratory because the sampling was not standardized. Thus, CA 15.3 should be interpreted as an adjunct to clinical and radiological assessment.

## 1. Introduction

Breast cancer is a biologically heterogeneous disease comprising clinically distinct subtypes defined primarily by hormone receptor (HR) and human epidermal growth factor receptor 2 (HER2) expression. These include HR-positive/HER2-negative, HER2-positive, and triple-negative breast cancer, with additional differentiation of HR-positive/HER2-negative tumors into luminal A-like and luminal B-like categories according to proliferative and clinicopathological characteristics. These subtypes differ in natural biology, patterns of metastatic spread, prognosis, and systemic treatment strategies. Within HR-positive/HER2-negative breast cancer, luminal A-like tumors generally exhibit lower proliferative activity and a more indolent clinical course, whereas luminal B-like tumors are characterized by higher proliferative activity that is commonly reflected by higher Ki-67 expression and are generally associated with more aggressive clinical behavior and a less favorable prognosis. These biological differences may influence metastatic disease, treatment response, and survival patterns [1].

Advanced breast cancer remains an incurable disease, with a median overall survival of approximately 2–3 years and a 5-year survival rate of about 25% [2]. The patterns of metastatic spread vary according to molecular subtype, with HR-positive/HER2-negative tumors generally having a more favorable prognosis than other subtypes, although persistent elevation of CA 15.3 has been associated with worse clinical outcomes [3,4]. In Peru and other Latin American countries, access to advanced imaging may be limited, making serum tumor markers more frequently used in routine clinical practice.

Biomarkers play a central role in breast cancer classification, therapeutic selection, prognostic assessment, and disease monitoring. Specifically, circulating biomarkers represent an additional, minimally invasive source of information regarding tumor burden and disease evolution [1]. Among these, cancer antigen 15.3 (CA 15.3) is a conventional serum tumor marker commonly elevated in patients with HR-positive/HER2-negative advanced breast cancer and has been associated with a higher tumor burden and shorter overall survival [5]. CA 15.3 is elevated in up to 80% of patients with metastatic disease and has historically been used to support treatment monitoring [6,7,8].

Although the American Society of Clinical Oncology (ASCO) and the 6th and 7th ESO–ESMO International Consensus Guidelines recognize CA 15.3 as a potential adjunct for monitoring treatment responses, current clinical practice guidelines do not recommend its isolated use for diagnosis or therapeutic decision-making. Instead, imaging studies remain the standard method for assessing disease progression [8,9,10].

Despite its frequent use in advanced breast cancer, the clinical interpretation of CA 15.3 remains controversial because it cannot replace radiological or clinical assessment. Moreover, real-world data specifically evaluating its prognostic association in HR-positive/HER2-negative advanced breast cancer remain limited in Latin American populations. In general, studies have examined both baseline and longitudinal CA 15.3 measurements. Serial CA 15.3 assessment has been evaluated for its ability to detect secondary metastatic disease, whereas studies in patients receiving systemic therapy, including CDK4/6 inhibitors, have demonstrated associations between increases in CA 15.3 and disease progression. Nevertheless, increases in marker expression may not consistently correspond to radiological progression or treatment failure, supporting its interpretation only in combination with clinical and imaging findings [11,12,13,14].

Therefore, this study aimed to evaluate the association between baseline and follow-up CA 15.3 levels, clinicopathological characteristics, and overall survival in patients with HR-positive/HER2-negative ABC treated at a single public oncologic institution in Peru. Although the preliminary findings from this cohort were previously presented as a conference abstract [15], the present full-length study substantially extends that preliminary report by incorporating detailed methodological characterization, adjusted multivariable survival analyses, effect estimates with confidence intervals, and a rigorous exploratory assessment of baseline/follow-up CA 15.3 patterns and their methodological limitations.

## 2. Materials and Methods

**Figure 1:**
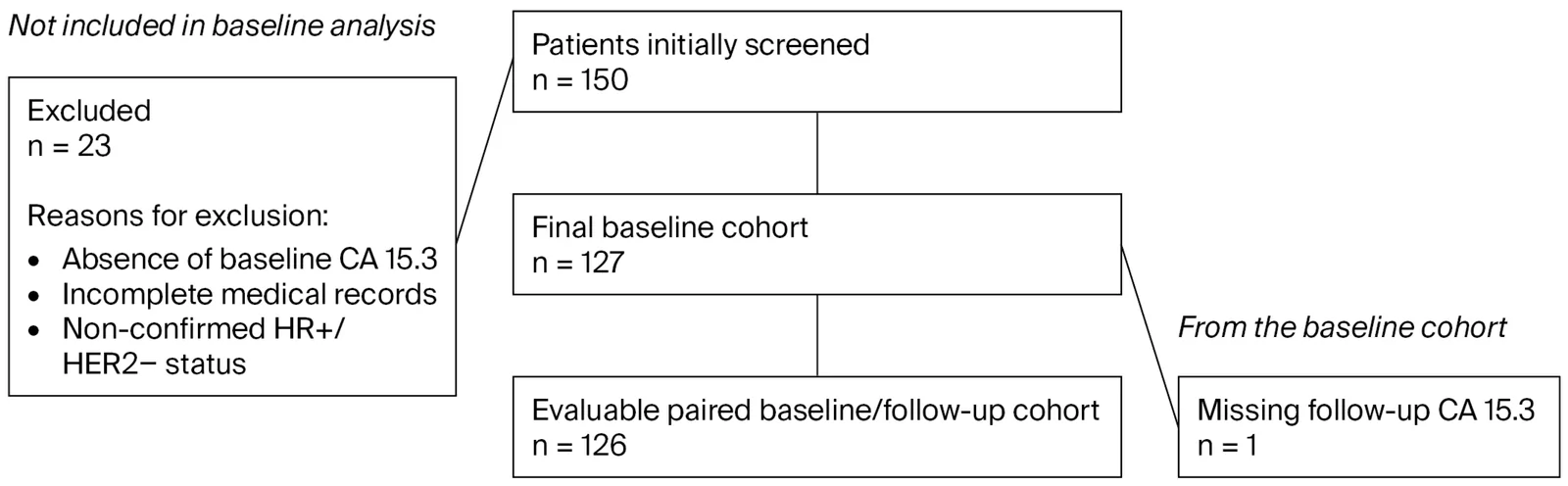
**Cohort selection flow diagram.** Selection of patients from initial screening to the baseline cohort and for the exploratory paired baseline/follow-up CA 15.3 cohort. HR, hormone receptor; HER2, human epidermal growth factor receptor 2.

Patients were eligible to be included in the overall study cohort if they had histologically confirmed HR-positive/HER2-negative advanced breast cancer, were treated at the Instituto Nacional de Enfermedades Neoplásicas (INEN) between 2018 and 2024, and had an available baseline CA 15.3 measurement obtained before the initiation of first-line systemic therapy. Patients with both de novo metastatic disease and metastatic recurrence after an initially non-metastatic breast cancer diagnosis were eligible. For the exploratory baseline/follow-up analysis, an additional evaluable CA 15.3 measurement during follow-up was required. Patients were excluded if they had no available baseline CA 15.3 measurement, incomplete medical records, or unconfirmed HR+/HER2− status. A cohort selection flow diagram is shown in Figure 1.

HR positivity was defined as estrogen receptor and/or progesterone receptor expression in ≥1% of tumor cells, and HER2 negativity was defined according to contemporaneous ASCO/CAP criteria. All tumors were hormone receptor-positive and HER2-negative. Luminal A-like and luminal B-like categories were assigned using an institutional immunohistochemistry-based surrogate classification according to the Ki-67 proliferation index. Ki-67 expression was assessed by routine immunohistochemistry at the INEN pathology laboratory and reported as the percentage of positively stained tumor cell nuclei (0–100%). A prespecified cutoff of 20% was used: tumors with Ki-67 ≤ 20% were classified as luminal A-like, whereas those with Ki-67 > 20% were classified as luminal B-like.

Serum CA 15.3 was measured using the Atellica IM CA 15-3 assay (Siemens Healthineers, Erlangen, Germany) on the Atellica IM Analyzer. The assay is a fully automated two-step sandwich immunoassay using direct chemiluminescent technology and monoclonal antibodies directed against the DF3 and 115D8 epitopes. According to the manufacturer, the upper limit of the normal reference interval is 32.4 U/mL, corresponding to the 99th percentile of measurements obtained from 399 apparently healthy women aged >18 years. Therefore, baseline CA 15.3 levels were classified as normal (<32.4 U/mL) or elevated (≥32.4 U/mL). This cutoff was prespecified according to the analytical reference interval and was not derived from outcome optimization in the present cohort. The same analytical platform and reference cutoff were used throughout the study period. Baseline CA 15.3 was defined as the measurement obtained before initiating first-line systemic therapy, and follow-up CA 15.3 measurements were obtained during routine clinical care and were not performed at predefined study-specific intervals. The follow-up value used in the exploration analysis corresponded to the last routinely available CA 15.3 measurement before the January 2025 data cutoff. Consequently, the interval between baseline and follow-up measurements varied among patients. In summary, a baseline and an evaluable follow-up measurement were available for 126 patients.

The median interval between baseline and the last available follow-up CA 15.3 measurement was 35 months (IQR, 21–63; range, 3–119 months). The clinical indication for the last CA 15.3 measurement was not systematically recorded and therefore, the follow-up measurement could not be consistently linked to a predefined radiological progression or treatment-modification event.

The distribution of continuous variables was assessed before selecting the corresponding statistical tests. In particular, Student’s *t*-test or analysis of variance (ANOVA) was used when parametric assumptions were considered appropriate, whereas the Mann–Whitney U test or Kruskal–Wallis test was used for non-normally distributed variables. For categorical variables, the Chi-square test was used when expected cell frequencies were adequate; when these assumptions were not met, Fisher’s exact test was applied. Variables for multivariable logistic regression were prespecified based on clinical relevance and biological plausibility rather than univariable statistical significance, and overall survival was additionally evaluated using Cox proportional hazards regression. A multivariable model was constructed by incorporating clinically relevant baseline prognostic factors, including age, ECOG performance status, de novo versus recurrent metastatic disease, luminal subtype, first-line treatment, number of metastatic sites, and metastatic location. The proportional hazards assumption was assessed using Schoenfeld residuals, and no significant violation was identified (global *p* = 0.48).

Overall survival (OS) was calculated from the date of systemic treatment initiation to the date of death or last follow-up. Survival curves were estimated using the Kaplan–Meier method and compared using the log-rank test, and a two-sided *p*-value < 0.05 was considered statistically significant. Statistical analyses were performed using R software version 4.3.2.

This study was conducted in accordance with the Declaration of Helsinki and was approved by the Institutional Review Board of the Instituto Nacional de Enfermedades Neoplásicas (protocol code INEN 25-46; approval date: 26 May 2025).

## 3. Results

### 3.1. Clinicopathological Characteristics of the Patients

A total of 127 female patients were included in the study. The median age was 55 years (IQR, 45–63), and 68% were postmenopausal. De novo metastatic disease was present in 42.5% of patients. Most patients had an ECOG performance status of 0–1 (93%), and luminal B was the predominant molecular subtype (75%). Regarding first-line treatment, 62% of patients received endocrine therapy, including 15% who were treated with ribociclib, whereas the remaining patients received chemotherapy. Based on the analysis of metastatic location, 43% had visceral metastases, 27% had bone-only metastases, and 30% had bone plus additional metastatic sites. Regarding the number of metastatic sites, 61%, 28%, and 10% of patients had one, two, and three or more metastatic sites, respectively.

**Figure 2:**
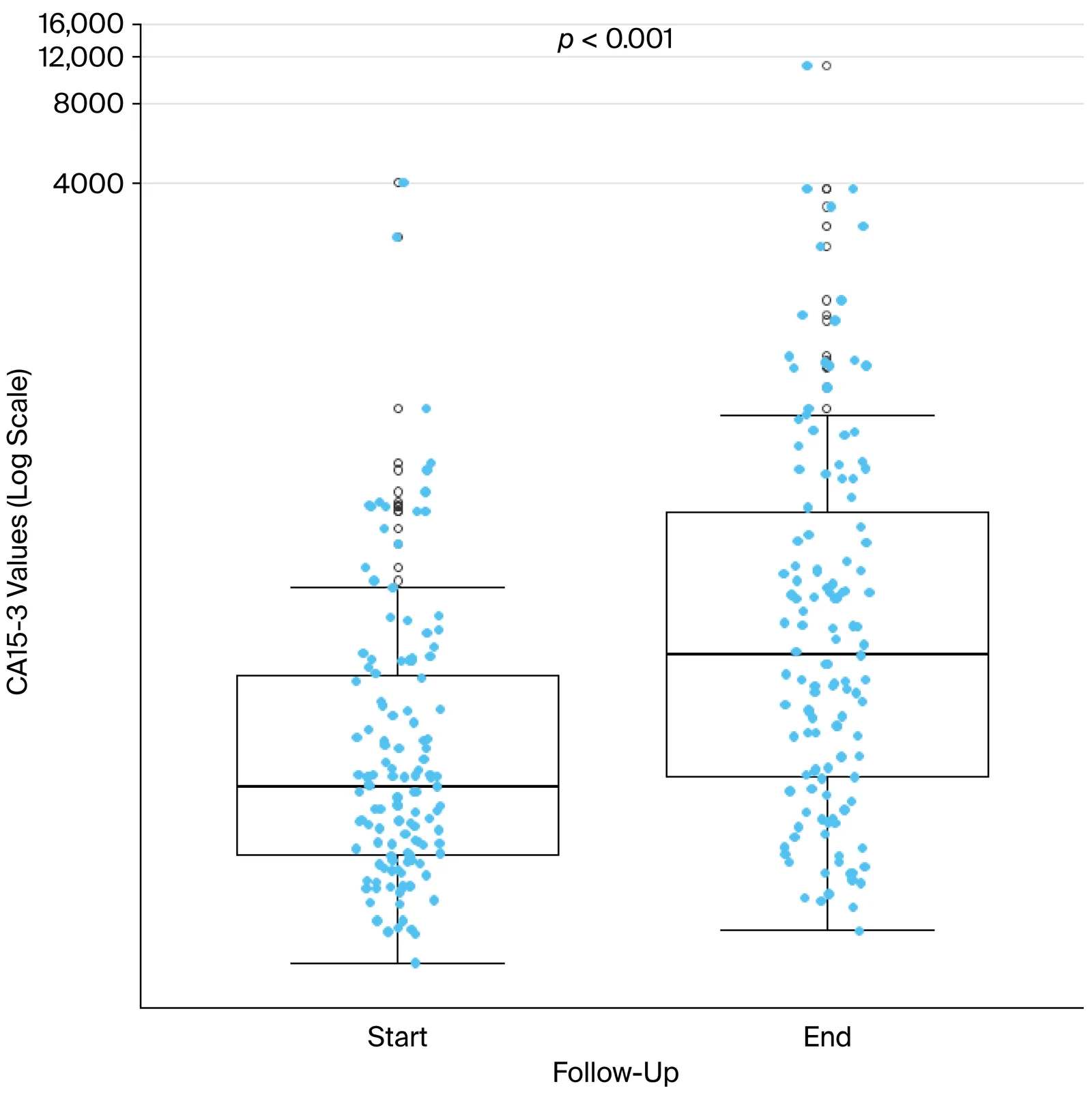
**CA 15.3 values at baseline and follow-up.** Baseline was defined as the measurement obtained before first-line systemic therapy, while follow-up represents the last routinely available measurement before the January 2025 data cutoff. The paired comparison was performed using the Wilcoxon signed-rank test. CA 15.3, cancer antigen 15.3; U/mL, units per milliliter.

With respect to tumor markers, 32% of patients had elevated baseline CA 15.3 levels, whereas 68% had elevated CA 15.3 levels during follow-up (median, 65.08 U/mL; *p* < 0.001) (Figure 2). Postmenopausal patients had significantly higher baseline CA 15.3 values than premenopausal (*p* = 0.011) women and were more likely to have elevated baseline CA 15.3 levels (*p* = 0.016) (Table 1, Table 2 and Table 3).

### 3.2. Factors Associated with Elevated Baseline CA 15.3 Levels

Multivariable logistic regression analysis demonstrated that postmenopausal status was independently associated with elevated baseline CA 15.3 levels (OR: 3.27; 95% CI, 1.33–9.03; *p* = 0.014). Similarly, patients with two or more metastatic sites had a significantly greater likelihood of presenting elevated baseline CA 15.3 levels than those with a single metastatic site (OR: 2.72; 95% CI, 1.24–6.10; *p* = 0.013) (Table 4).

### 3.3. Baseline and Follow-Up CA 15.3 Levels

**Figure 3:**
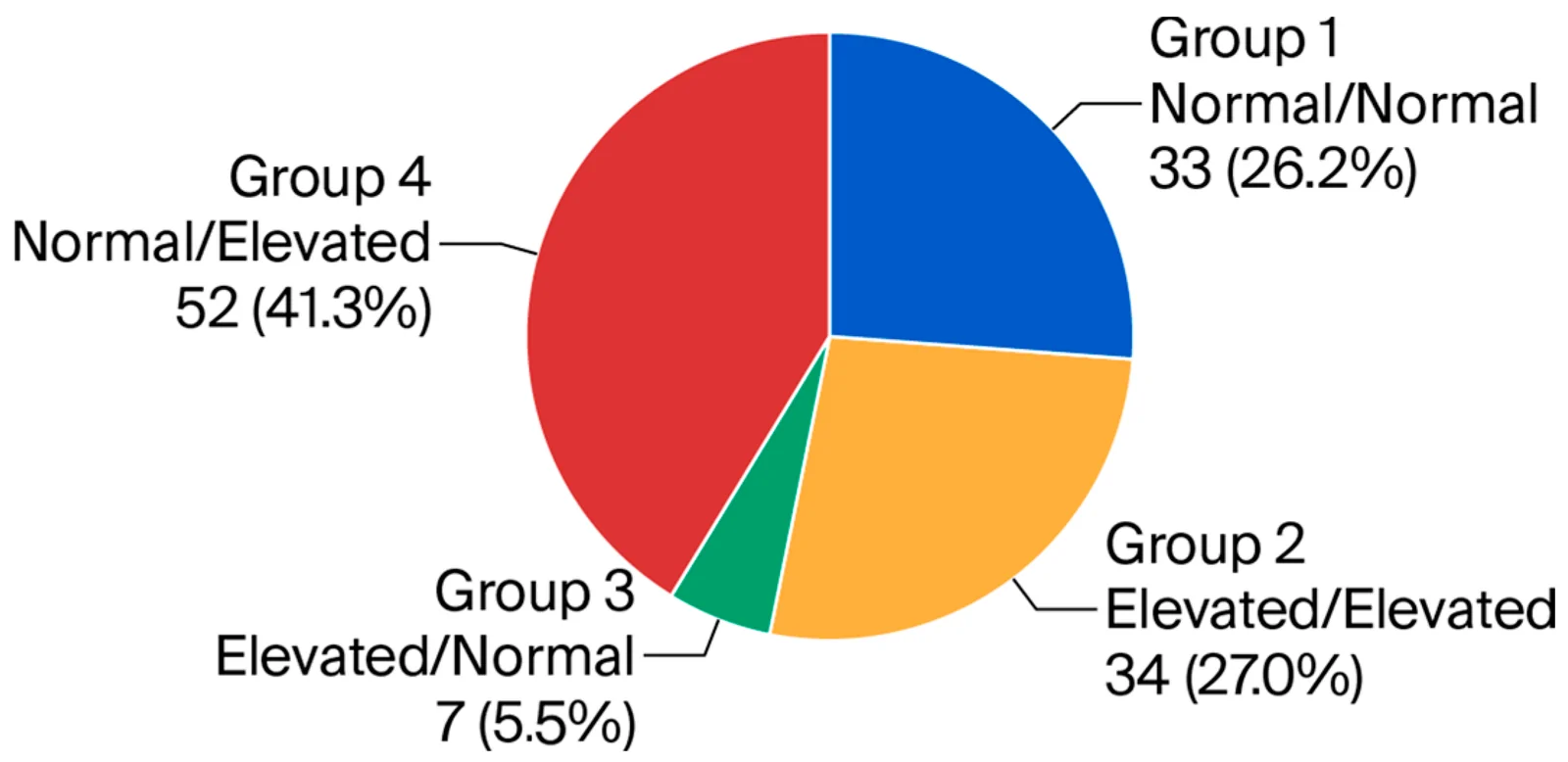
**Distribution of 126 evaluable patients according to baseline and follow-up CA 15.3 patterns.** Normal CA 15.3 expression was defined as <32.4 U/mL, while elevated CA 15.3 expression was defined as ≥32.4 U/mL. Group 1, normal/normal; Group 2, elevated/elevated; Group 3, elevated/normal; Group 4, normal/elevated.

According to baseline and follow-up CA 15.3 measurements, patients were classified into four descriptive and exploratory groups: normal/normal (26.2%), elevated/elevated (27.0%), elevated/normal (5.5%), and normal/elevated (41.3%) (Figure 3).

According to multivariable logistic regression, postmenopausal status was independently associated with elevated baseline CA 15.3 levels compared with premenopausal status (OR: 3.27; 95% CI, 1.33–9.03; *p* = 0.014). Similarly, patients with two or more metastatic sites had greater odds of having elevated baseline CA 15.3 levels than those with a single metastatic site (OR: 2.72; 95% CI, 1.24–6.10; *p* = 0.013) (Table 4).

Figure 3 shows the distribution of evaluable patients by group according to baseline and follow-up CA 15.3 levels. There were 33 (26.2%) patients who had normal CA 15.3 levels at baseline and follow-up, while 34 (27.0%) patients had elevated CA 15.3 at baseline and follow-up. In contrast, seven (5.5%) patients had elevated CA 15.3 levels at baseline and normal expression at follow-up, and 52 (41.3%) patients had normal CA 15.3 levels at baseline and elevated expression at follow-up.

### 3.4. Overall Survival

**Figure 4:**
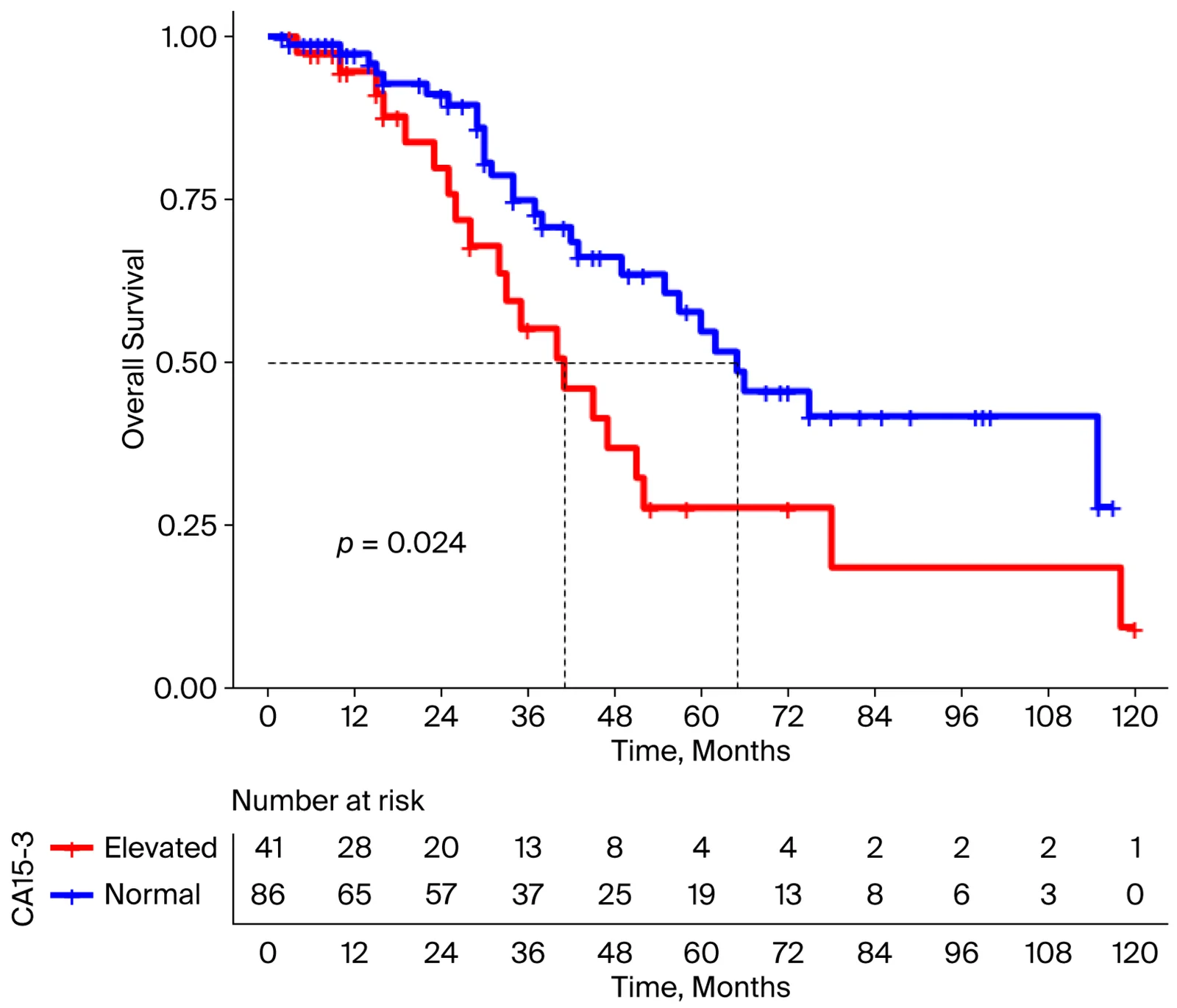
**Estimated overall survival curves according to baseline CA 15.3 levels in all patients.** Kaplan–Meier estimates of overall survival according to baseline CA 15.3 status. *p*-values were calculated using the log-rank test. CA 15.3 <32.4 U/mL was classified as normal, while ≥32.4 U/mL was considered elevated.

After a median follow-up of 30 months (range, 2–120), the median OS for the overall cohort was 55 months (95% CI, 43–78). The estimated OS rates at 12, 36, and 60 months were 97% (95% CI, 93–100%), 69% (95% CI, 60–80%), and 45% (95% CI, 35–59%), respectively (Table 5). Patients with normal baseline CA 15.3 expression had significantly longer OS than those with elevated baseline levels (median OS, 65 vs. 41 months; log-rank *p* = 0.024) (Figure 4). Specifically, the estimated OS among patients with normal baseline CA 15.3 levels at 12, 36, and 60 months was 97% (95% CI, 94–100), 75% (95% CI, 65–87), and 55% (95% CI, 42–72), respectively, compared with 95% (95% CI, 88–100), 55% (95% CI, 39–78), and 28% (95% CI, 14–54), respectively, among patients with elevated baseline CA 15.3.

Among patients with luminal B-like disease, those with normal baseline CA 15.3 expression also had significantly longer OS than those with elevated baseline levels (log-rank *p* = 0.032). Median OS was 65 months (95% CI, 55–NE) in the normal CA 15.3 group and 41 months (95% CI, 32–NE) in the elevated CA 15.3 group, where NE indicates that the upper confidence limit was not estimable.

**Figure 5:**
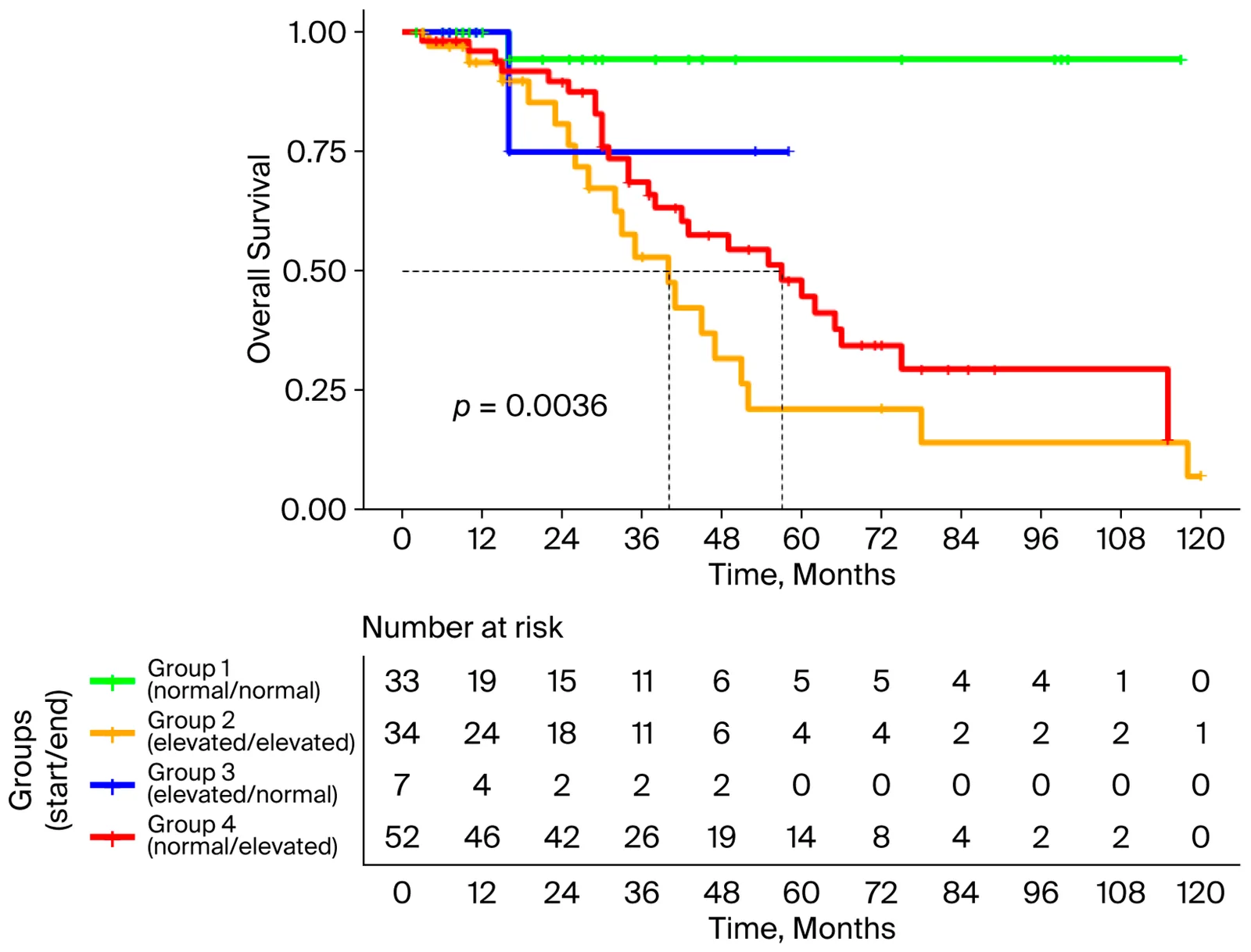
**Estimated overall survival according to baseline and follow-up CA 15.3 patterns.** Exploratory Kaplan–Meier analysis according to baseline/follow-up CA 15.3 patterns among 126 evaluable patients. Group 1, normal/normal (n = 33); Group 2, elevated/elevated (n = 34); Group 3, elevated/normal (n = 7); Group 4, normal/elevated (n = 52). The analysis should be considered exploratory because follow-up measurements were not obtained at standardized intervals.

In the exploratory longitudinal analysis, OS differed significantly across the four CA 15.3 patterns (log-rank *p* = 0.0036). Patients with persistently elevated CA 15.3 levels (elevated/elevated) and those who developed elevated levels during follow-up (normal/elevated) had poorer survival than patients with persistently normal CA 15.3 levels (Table 6 and Figure 5).

After adjusting for clinically relevant baseline prognostic factors (age, ECOG, de novo metastasis, luminal subtype, first-line treatment, number of metastases, and metastatic location), elevated baseline CA 15.3 was associated with OS (adjusted HR: 3.42; 95% CI 1.63–7.19; *p* = 0.001). In contrast, de novo metastatic presentation was not independently associated with OS compared with recurrent metastatic disease (adjusted HR: 0.79; 95% CI, 0.39–1.60; *p* = 0.516).

## 4. Discussion

Approximately one-third of patients had elevated baseline CA 15.3 levels, a proportion lower than that reported in several metastatic breast cancer series. Differences in assay thresholds, measurement timing, tumor burden, and population characteristics may partly explain this variability. In international cohorts, CA 15.3 has shown greater sensitivity than other tumor markers such as CEA (~40%) and CA 125 (~20%) for detecting metastatic breast cancer, with the latter being especially sensitive in peritoneal or gynecological (ovarian) metastases due to breast cancer [11]. In a retrospective analysis of 743 patients, CA 15.3 kinetics showed greater sensitivity than CEA for detecting metastatic disease (55.6% vs. 40.6%), while the combination of both markers increased sensitivity to 66.3% [12]. Regarding subtypes, increased CA 15.3 expression is less frequent in non-HR-positive tumors. There is an association between HR-positive status and a better prognosis in patients with elevated CA 15.3 prior to therapy for advanced disease compared with HR-negative tumors [11]. Additionally, in patients with tumor marker elevation during the last six months before death, subtype distribution patterns were similar to those observed at diagnosis (*p* < 0.0001).

Several studies, particularly those on luminal subtypes, have reported frequent CA 15.3 elevation, with rates ranging from 37% to over 90%, depending on the methodology and cut-off values used. In LATAM, retrospective series consistently report elevations in more than half of the patients, which aligns with our findings [3]. In contrast, elevation was less frequent in basal (74.1%) and non-basal (71.4%) triple-negative subtypes, with a statistically significant difference (*p* < 0.001). In the study by Yerushalmi et al., CA 15.3 positivity was defined using a cutoff of >28 U/mL. In contrast, other studies have reported more variable values, which could be explained by methodological differences or by differences in the cutoff points used. For example, a retrospective study analyzing advanced-stage disease (n = 284) reported elevated CA 15.3 frequencies of 65.9% in patients with luminal A, 68.6% in luminal B tumors, and 62.2% in HR-positive/HER2-positive ABC tumors with a CA 15.3 cutoff of > 31.3 U/mL [6]. Similarly, in a cohort of patients evaluated at metastatic relapses (n = 272), elevated CA 15.3 was observed in 69% of HR-positive/HER2-negative ABC tumors and 56% of HR-positive/HER2-positive ABC tumors with a CA 15.3 cutoff of >100 U/mL [13]. On the other hand, a study that included ABC patients (n = 730) found elevated CA 15.3 levels in 48% of cases with luminal A tumors, 44% of HR-positive/HER2-positive ABC patients, and 37% of luminal B patients [14]. These data suggest that, although luminal subtypes tend to show more frequent elevation of CA 15.3 compared to non-luminal ones, the sensitivity of this marker in the metastatic context varies substantially between studies. In LATAM, two retrospective studies reported that CA 15.3 was elevated in more than 50% of cases with metastatic disease [16,17]. Additionally, luminal tumors are also predominant in Peru. In the PEGEN-BC cohort (n = 1943), HR-positive/HER2-negative disease was the most frequent subtype, accounting for 52.4% of cases, followed by HR-positive/HER2-positive tumors at 18.7% [18].

The definition of CA 15.3 elevation varies widely across studies, from 15% above baseline to thresholds based on the upper limit of normal. In our study, the mean increase was 33 U/mL among patients with elevated baseline values, which is comparable to previous reports [19,20]. This suggests that our cutoff remains clinically relevant, even in a Peruvian population. A large trial on newly diagnosed breast cancer reported that patients with elevated baseline CA 15.3 (>30 U/mL) were at a higher risk of developing de novo or secondary metastatic disease [21], and our findings are consistent with this observation, highlighting the persistently elevated CA 15.3 expression during follow-up and its association with shorter survival. In Peru, where many patients present with advanced disease at diagnosis, this marker may help identify patients with a less favorable prognosis.

In our cohort, persistent or newly developed CA 15.3 elevation was associated with poorer OS in the exploratory longitudinal analysis, even in patients with normal baseline levels. These findings are consistent with international series where CA 15.3 has been expressed as an independent prognostic factor, notably in HR-positive/HER2-negative tumors. In localized disease, one cohort demonstrated an absolute reduction of 19.1% in 5-year OS (90.8% vs. 71.7%; *p* < 0.001) in the presence of elevated levels [22]. In metastatic disease, several studies have documented reductions of 10 to 24 months in median OS (*p* = 0.002–0.02) in patients with high CA 15.3 expression at diagnosis or during follow-up, which was associated with a higher tumor burden [23]. Therefore, its sustained increase may precede the radiological detection of recurrence by months.

Our cohort had a higher proportion of de novo metastatic disease and a slightly lower postmenopausal percentage than several international series. The reasons underlying this difference were not evaluated in the present study and may involve multiple healthcare- and disease-related factors [23]. In the prospective study by Sottotetti et al., baseline elevations of CA 15.3 and CEA were observed in 81% and 50% of patients, respectively. The cohort (n = 142) included patients receiving a CDK4/6 inhibitor, predominantly palbociclib, combined with endocrine therapy, with no significant correlations with baseline characteristics (primary vs. secondary endocrine resistance, metastatic location, and line of treatment). A correlation was observed between an increase in CA 15.3 of >20% and the change between the best response and progressive disease (PD), a change of 29 U/mL (5.5–106.3, *p* < 0.001) [24]. The proportion of postmenopausal patients was higher in Sottotetti et al.’s study than in our cohort (81% vs. 68.5%), whereas the prevalence of visceral metastases was similar (43% vs. 42.5%). In contrast, our study reported a higher incidence of de novo disease (42.5% vs. 18%). It should be noted that a small percentage of our patients (15%) used a CDK 4/6 inhibitor (ribociclib) as first-line therapy; however, the majority received aromatase inhibitors as monotherapy. A study of ABC patients treated with fulvestrant (n = 67) observed that in patients with disease progression, CA 15.3 levels significantly increased after four months of treatment. However, it was also noted that CA 15.3 levels may rise in patients who ultimately experience clinical benefit [25].

The association between elevated CA 15.3 and poorer OS may partly reflect greater disease burden, as baseline elevation was more common among patients with multiple metastatic sites. Persistent or newly developed elevation could similarly accompany sustained or increasing tumor burden. Despite this, the baseline association remained significant after adjustment for established prognostic factors, including metastatic number and site. The follow-up patterns remain hypothesis-generating because biomarker sampling was not standardized and CA 15.3 changes were not prospectively correlated with radiological progression or response criteria.

In our cohort, patients with normal baseline CA 15.3 (65 vs. 41 months, *p* = 0.024) expression and a normal baseline level in the luminal B subtype (*p* = 0.032) had longer OS compared to those with elevated CA 15.3 levels. Although previous studies have evaluated CA 15.3 as an adjunct to treatment monitoring, our study was not designed to evaluate its ability to detect radiological progression or predict treatment responses.

Regarding the use of chemotherapy in HR-positive/HER2-negative ABC, previous studies found a significant relationship between disease response and variations in CA 15.3 levels [26], but with notable individual discrepancies. Another study reported that changes in CA 15.3 levels at 8 and 12 weeks after starting treatment appeared to correlate with treatment response in patients who had elevated markers prior to initiating therapy [27]. Conversely, another retrospective study including patients receiving radiotherapy in addition to systemic therapy (n = 118) reported that CA 15.3 did not provide prognostic information; instead, factors such as the extent of metastases, lung/pleural involvement, ER status, and anemia requiring blood transfusion (all *p* < 0.01) had a stronger influence on OS than CA 15.3 levels [28]. Our study evaluated prognostic associations and does not directly establish treatment-response monitoring utility because radiological responses, predefined progression criteria, and progression-free survival were not systematically analyzed in relation to CA 15.3 changes.

Some studies suggest that CA 15.3 may be more strongly associated with bone metastases [29]. In contrast, we did not observe such correlations in our population. Instead, survival was more clearly linked to the number of metastatic sites (≥ 2). This aligns with the frequent presentation of high tumor burden in our public institution. Additionally, population characteristics may influence outcomes: local reports in Peruvian cohorts with HR-positive/HER2-negative [30] and HER2-positive [31] ABC have described a higher incidence of de novo disease, a higher metastatic burden, and rapidly symptomatic (“aggressive”) disease, with treatment-access challenges observed in routine public-sector practice.

Regarding the four-group analysis, these findings should be considered hypothesis-generating and require prospective validation. Patients whose CA 15.3 levels were normalized during follow-up (Group 3) represent a subgroup with unclear clinical implications. In addition, Group 3 included seven patients and one death only; therefore, survival estimates for this group should not be interpreted as evidence of a distinct prognostic pattern. Currently, no guidelines address how these cases should be managed, underscoring the need for further research in larger cohorts.

The role of CA 15.3 in follow-up remains debated. Some studies combining tumor marker elevation with advanced imaging detected asymptomatic metastases in over 60% of cases, suggesting that reproducible increases should prompt further imaging [32]. This highlights its potential utility, even in real-world, resource-constrained settings, as monitoring serum CA 15.3 levels is relatively inexpensive, accessible, and simple. Baseline CA 15.3 was associated with overall survival in this cohort, but its incremental prognostic value beyond established clinical factors requires further validation. In fact, in those studies, there was no significant relationship between metastatic site and either CEA (*p* = 0.93) or CA 15.3 (*p* = 0.20) levels [33].

Our study has several limitations, including its retrospective design, small sample, reliance on a single biomarker, therapeutic heterogeneity (almost 50% received aromatase inhibitor alone), and relatively short follow-up. Treatment exposure was heterogeneous, and detailed longitudinal information on treatment duration, subsequent treatment lines, treatment modifications, endocrine sensitivity/resistance, and radiological responses was not uniformly available. Using the last available CA 15.3 measurement may introduce informative bias since the timing and frequency of testing may be related to disease course, follow-up duration, treatment changes, or progressive disease. Therefore, residual confounding related to treatment selection, follow-up measurement, and treatment evolution cannot be interpreted causally. The study spans 2018–2024, a period during which systemic treatment standards and access to CDK4/6 inhibitors evolved. Consequently, calendar-era effects and changes in treatment availability may have influenced survival and follow-up biomarker patterns. Additionally, the relatively small cohort and limited number of outcome events restrict the precision of subgroup estimates; findings derived from follow-up CA 15.3 patterns should therefore be considered exploratory and require validation in larger cohorts. Potentially relevant prognostic variables, including disease-free interval, endocrine sensitivity/resistance, detailed prior adjuvant therapy, duration and number of systemic treatment lines, treatment modifications, and radiological responses, were not uniformly available and could not be incorporated into the analysis.

Our findings provide real-world data from an underrepresented Peruvian public-sector population; however, the single-center design and local patterns of treatment access limit external validity, and the results should primarily be interpreted in the context of similar resource-constrained healthcare settings [15]. Randomized controlled trials maximize internal validity through controlled eligibility criteria and randomized treatment allocation, whereas real-world studies provide greater representation of routine clinical practice but remain more susceptible to confounding and selection bias.

## 5. Conclusions

In this real-world Peruvian cohort of patients with HR-positive/HER2-negative advanced breast cancer, elevated baseline CA 15.3 expression was associated with greater metastatic burden and independently associated with shorter overall survival after adjustment for clinically relevant prognostic factors (adjusted HR: 3.42; 95% CI, 1.63–7.19; *p* = 0.001). The baseline/follow-up CA 15.3 patterns should be interpreted as exploratory because the measurements were obtained at non-standardized intervals and may be affected by time-related and informative-measurement biases. CA 15.3 should therefore be interpreted as an adjunct to clinical and radiological assessment. Thus, prospective multicenter studies with predefined biomarker sampling schedules and appropriate time-dependent analyses are warranted.

## Figures and Tables

**Table 1:** Clinicopathological characteristics of the patients included in the study.

	n = 127
Age, years	
Median [IQR]	55 [45–63]
ECOG	
0–1	119 (93.7%)
2	8 (6.3%)
De novo metastases	
Yes	54 (42.5%)
No	73 (57.5%)
Luminal subtype	
Luminal A-like	25 (19.6%)
Luminal B-like	95 (74.8%)
Not evaluable	7 (5.6%)
First-line treatment	
Endocrine therapy (aromatase inhibitor)	60 (47.2%)
Endocrine therapy + CDK 4/6 inhibitor (ribociclib)	19 (15.0%)
Chemotherapy	48 (37.8%)
Number of metastases	
1	78 (61.4%)
2	36 (28.3%)
≥3	13 (10.3%)
Site of metastases	
Visceral	54 (42.5%)
Bone and additional metastatic sites	39 (30.7%)
Bone only	34 (26.8%)
Baseline values of CA 15.3, U/mL	
Median [IQR]	20.3 [11.1–54.83]
Baseline CA 15.3 level, n = 127	
Normal, <32.4 U/mL	86 (67.7%)
Elevated, ≥32.4 U/mL	41 (32.3%)
Follow-up CA 15.3 values, U/mL	
Median [IQR]	65.08 [22.06–234]
Follow-up CA 15.3 level, n = 126	
Normal, <32.4 U/mL	40 (31.7%)
Elevated, ≥32.4 U/mL	86 (68.3%)
One patient did not have an evaluable follow-up measurement	

CDK: cyclin-dependent kinase; IQR: interquartile range; U/mL: units per milliliter.

**Table 2:** Tumor marker CA 15.3 levels according to menopausal status.

	Menopausal Status (%)		*p*-Value
	Premenopausal n = 40	Postmenopausal n = 87	
Baseline CA 15.3 value, U/mL			
Median [IQR]	12.6 [9.97–22.29]	23.37 [13.37–61.41]	0.011
Baseline CA 15.3 level			
Normal, <32.4 U/mL	33 (82.5%)	53 (60.9%)	
Elevated, ≥32.4 U/mL	7 (17.5%)	34 (39.1%)	0.016

IQR: interquartile range, U/mL: units per milliliter.

**Table 3:** Tumor marker CA 15.3 levels according to number of metastases.

	Number of Metastases (%)		*p*-Value
	1 n = 78	≥2 n = 49	
Baseline CA 15.3 value, U/mL			
Median [IQR]	18.87 [10.67–30.69]	29.15 [12.38–65.28]	0.088
Baseline CA 15.3 level			
Normal, <32.4 U/mL	59 (75.6%)	27 (55.1%)	
Elevated, ≥32.4 U/mL	19 (24.4%)	22 (44.9%)	0.016

IQR: interquartile range, U/mL: units per milliliter.

**Table 4:** Estimates of the effect of the studied characteristics on the likelihood of a high baseline CA 15.3 level.

		Univariate			Multivariable	
	OR	95% CI	*p*-Value	OR	95% CI	*p*-Value
Menopausal status						
Premenopausal	Ref.			Ref.		
Postmenopausal	3.02	[1.26–8.14]	0.019	3.27	[1.33–9.03]	0.014
Number of metastases						
1	Ref.			Ref.		
≥2	2.53	[1.18–5.49]	0.017	2.72	[1.24–6.10]	0.013

OR: odds ratio; CI: confidence interval; Ref.: reference.

**Table 5:** Overall survival (OS) according to baseline CA 15.3 levels.

Population	OS, Months (95% CI)			Median OS, Months (95% CI)
	12	36	60	
Overall cohort	97% (93–100)	69% (60–80)	45% (35–59)	55 (43–78)
Normal baseline CA 15.3	97% (94–100)	75% (65–87)	55% (42–72)	65
Elevated baseline CA15.3	95% (88–100)	55% (39–78)	28% (14–54)	41

**Table 6:** Overall survival (OS) according to groups (baseline/follow-up CA 15.3 levels).

	N (Events)	OS, Months			*p*-Value
		12	36	60	
Evaluable patients	126 (48)	97%	69%	45%	-
Group (baseline/follow-up)					0.0036
Group 1 (normal/normal)	33 (1)	100%	94%	94%	
Group 2 (elevated/elevated)	34 (19)	94%	53%	21%	
Group 3 (elevated/normal)	7 (1)	100%	75%	-	
Group 4 (normal/elevated)	52 (27)	96%	69%	45%	

Note: The overall cohort included 127 patients; one patient without an evaluable follow-up CA 15.3 measurement was excluded from the exploratory longitudinal group analysis.

## Data Availability

The data are not publicly available because they contain confidential clinical information. De-identified data may be available from the corresponding author upon reasonable request and subject to approval by the Institutional Review Board and the Instituto Nacional de Enfermedades Neoplásicas (INEN). Institutional information and contact channels are available through the official INEN portal at https://www.gob.pe/inen (accessed on 25 August 2026).

## Abbreviations

The following abbreviations are used in this manuscript:

ABC	Advanced breast cancer
CA 15.3	Cancer antigen 15.3
CDK 4/6	Cyclin-dependent kinase 4/6
CEA	Carcinoembryonic antigen
CI	Confidence interval
CPGs	Clinical practice guidelines
ECOG	Eastern Cooperative Oncology Group
HR	Hormone receptor
INEN	Instituto Nacional de Enfermedades Neoplásicas
IQR	Interquartile range
LATAM	Latin America
NE	Not estimable
OR	Odds ratio
OS	Overall survival
RCTs	Randomized controlled trials
RWD	Real-world data
TM	Tumor marker
